# Clinical values of serum Semaphorin 4D (Sema4D) in medication‑related osteonecrosis of the jaw

**DOI:** 10.1186/s40001-023-01095-6

**Published:** 2023-03-30

**Authors:** Hong Mu, Ying Pang, Lili Liu, Jingbo Liu, Chunsheng Liu

**Affiliations:** 1 Dental Clinic, Cangzhou Central Hospital, Xinhua West Road, Cangzhou, 061000 Hebei China; 2 General Department, Cangzhou Stomatological Hospital, Xinhua West Road, Cangzhou, 061000 Hebei China; 3 Department of Oral and Maxillofacial Surgery, Cangzhou People’s Hospital, Qingchi Avenue, Cangzhou, 061000 Hebei China

**Keywords:** Bisphosphonates, MRONJ, Sema4D, Surgery

## Abstract

**Background:**

Bisphosphonates (BPs) are widely used in clinical practice to prevent and treat bone metabolism-related diseases. Medication-related osteonecrosis of the jaw (MRONJ) is one of the major sequelae of BPs use. Early prediction and intervention of MRONJ are of great significance.

**Methods:**

Ninety-seven patients currently on treatment with BPs or with a history of BPs usage and 45 healthy volunteers undergoing dentoalveolar surgery were included in this study. Participants' serum Semaphorin 4D (Sema4D) levels were measured and analyzed before participants underwent surgery (T0) and after a 12-month follow-up (T1). Kruskal–Wallis test and ROC analysis were used to examine the predictive effect of Sema4D on MRONJ.

**Results:**

Sema4D levels in serum of patients corresponding to confirmed MRONJ were significantly lower at both T0 and T1 time points compared to non-MRONJ and healthy controls. Sema4D has a statistically predictive effect on the occurrence and diagnosis of MRONJ. Serum Sema4D levels were significantly reduced in MRONJ class 3 patients. MRONJ patients who received intravenous BPs had significantly lower Sema4D levels than those who received oral BPs.

**Conclusion:**

Serum Sema4D level has predictive value for the onset of MRONJ in BPs users within 12 weeks after dentoalveolar surgery.

## Background

Bisphosphonates (BPs) are stable analogs of pyrophosphate [1]. BPs are widely used in clinical practice for the prevention and treatment of bone metabolism-related diseases such as osteoporosis, multiple myeloma, osteitis deformans, bone metastases from malignant tumors, and tumor-derived hypercalcemia [2]. BPs inhibit the destruction of bone and control the bone metastasis of malignant tumors [3]. However, the deposition of BPs in bone cannot be completely metabolized [4]. Osteonecrosis of the jaw associated with BPs can occur many years later due to trauma, tooth extraction, etc. [5]. Therefore, the number of patients with BPs-related osteonecrosis of the jaw and patients with potential osteonecrosis risk is huge [6].

Medication-related osteonecrosis of the jaw (MRONJ) is a rare clinical disease that mainly occurs in patients with osteoporosis, bone metastases and other bone-destructive diseases who receive bisphosphonate therapy [7, 8]. The pathogenesis of MRONJ remains unclear [9]. Accumulating evidence has demonstrated that MRONJ may be associated with imbalances in bone remodeling, inhibition of angiogenesis, inflammatory response to infection, and soft tissue toxicity [9, 10]. Most of the current research shows that the treatment plan of MRONJ should comprehensively consider the disease stage and the patient's systemic condition [11]. Perioperative use of antibacterial mouthwash and systemic antibiotic therapy are effective measures to prevent the occurrence of MRONJ after tooth extraction [12].

Semaphorins are a newly discovered family of proteins with common domains that are widely present in organisms [13]. They have attracted widespread attention due to their bidirectional regulation of osteoclasts and osteoblasts [14]. It has been reported that some sema family proteins and their receptors are involved in the regulation of bone remodeling [15]. Semaphorin 4D (Sema4D) directly or indirectly affects the expression, differentiation and migration of osteoblasts and osteoclasts, thereby inhibiting or promoting the process of bone remodeling [16]. Sema4D expression is increased in canonical receptor activator of nuclear kappa-B (RANKL)-mediated osteoclast differentiation [17]. It was found that Sema4D protein expression was significantly reduced in MRONJ model tissues by immunohistochemical staining in animal models [13].

In order to further verify the clinical value of Sema4D in MRONJ and provide a basis for early warning and diagnosis of MRONJ, we study the clinical significance of Sema4D in the early diagnosis of MRONJ in this work.

## Methods

### Study design

**Fig. 1:**
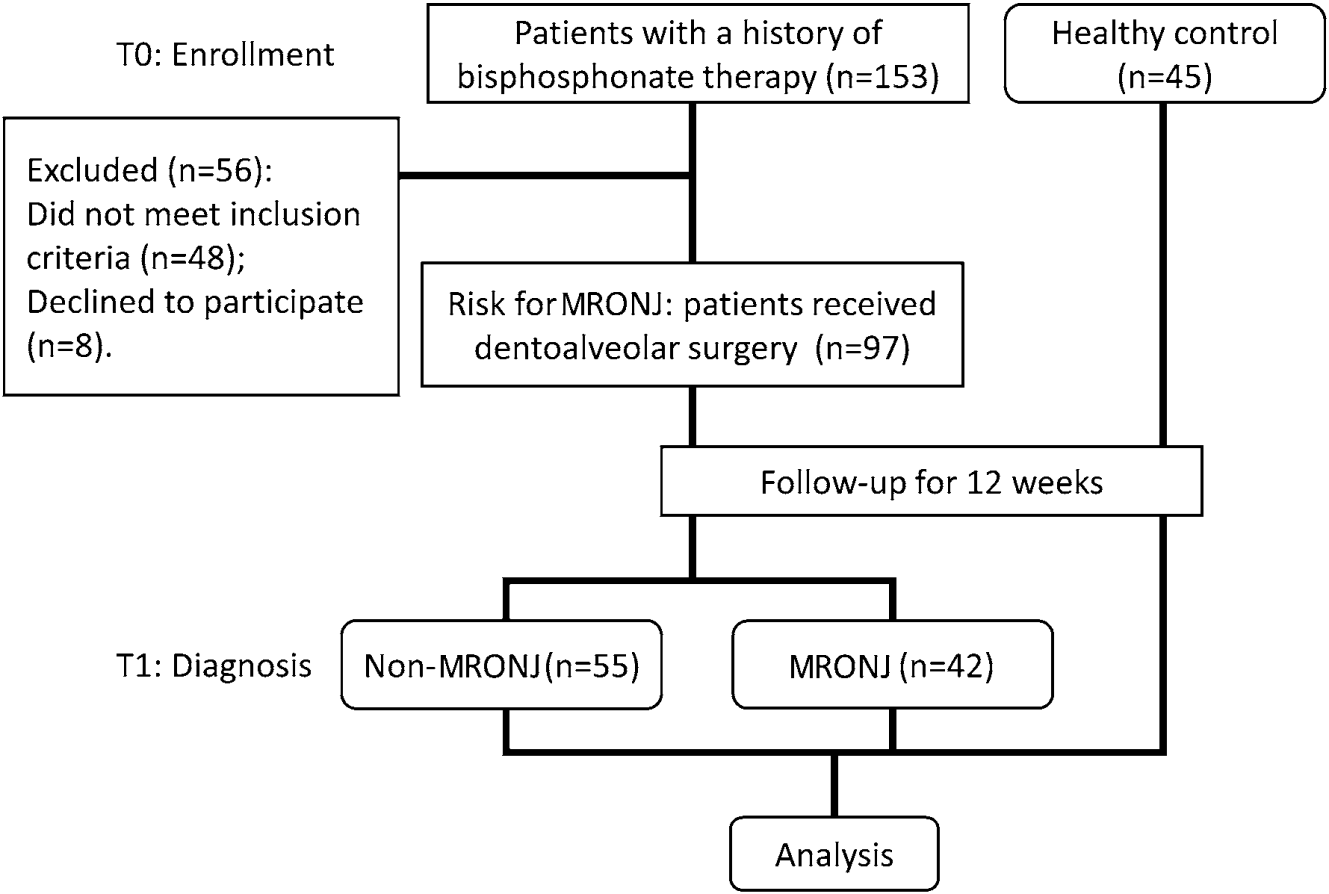
Study design

A total of 153 patients who had currently on treatment with oral bisphosphonates for more than 2 years or had received at least two intravenous bisphosphonate injections were preparing for dentoalveolar surgery. Eight patients declined to participate in the study, 28 patients were experiencing MRONJ, 13 patients had previous MRONJ, and 7 patients were excluded for other reasons. Therefore, 97 patients at risk for MRONJ were included. After 12 weeks of follow-up, 55 patients had no confirmed MRONJ to the follow-up endpoint, and 42 patients had confirmed MRONJ. The time point at which patients at risk for MRONJ were included in the study was defined as T0. The time point of the study endpoint after the end of 12-month follow-up was defined as T1. In addition, we selected 45 healthy patients to detect serum Sema4D levels at T0 and T1 time points (Fig. 1). The study was approved by the ethics committee of Cangzhou Central Hospital (#2021-189-02(Z)), and the participants signed written informed consent.

### MRONJ diagnosis

Diagnostic criteria for MRONJ: current or previous history of bisphosphonate therapy; osteonecrosis of the jaw for more than 8 weeks without improvement; no history of radiotherapy to the head and neck. Patients meeting the above three conditions at the same time can be diagnosed as MRONJ.

### Staging of MRONJ

MRONJ is divided into risk period, stage 0, stage 1, stage 2 and stage 3 clinically. Patients at risk of MRONJ were asymptomatic and without osteonecrosis. Patients with MRONJ stage 0 mainly present with no osteonecrosis or bone exposure, and only non-specific symptoms. Patients with MRONJ stage 1 mainly present with osteonecrosis or bone exposure, without clinical symptoms and signs of infection. Patients with MRONJ stage 2 had osteonecrosis or bone exposure with focal infection. Patients with MRONJ stage 3 have osteonecrosis or exposed bone with painful infection. In addition, stage 3 MRONJ should also include one or more of the following manifestations: pathological fracture, extraoral fistula, and jaw with lesions beyond the alveolar bone.

### Participants

All participants in this study received elective dentoalveolar surgery in our hospital, including frenulum correction, alveolar bone revision, and oro-antral fistula repair. Inclusion criteria: (1) Patients who underwent alveolar surgery in our hospital; (2) Patients who received oral BPs for at least 2 years or at least two intravenous injections of BPs; (3) No head and neck radiation therapy; (4) Patients without jaw metastatic disease. Exclusion criteria: (1) Patients who already suffered from MRONJ at the time of recruitment. (2) Patients with current evidence of MRONJ (exposed bone or bone that can be explored through the formation of intraoral or extraoral fistulas in the maxillofacial region); (3) Patients with serious cardiovascular and cerebrovascular and systemic diseases, allergies, and mental illnesses.

The 97 patients at risk for MRONJ were included in this study. After 12 weeks of follow-up, 55 patients had no confirmed MRONJ to the follow-up endpoint, and 42 patients had confirmed MRONJ. Therefore, they were divided into non-MRONJ group (n = 55) and MRONJ group (n = 42). In addition, we also recruited 45 healthy people without MRONJ risk as a healthy control group (n = 45).

### Intervention

In order to detect the concentration of Sema4D in the serum of different patients in different periods, we collected 5 mL of fasting peripheral venous blood from each patient at T0 and T1 time points respectively. Patients were asked to fast for eight hours before the blood collection, which took place from 6:00 am to 9:00 am. The blood collection process was completed by experienced nurses using vacuum blood collection tubes according to the hospital's blood collection procedures. Each patient was required to have two tubes of blood collected at each blood draw for research and storage. Blood collected was centrifuged at 3,000 g for 10 min to obtain patient serum immediately on the same day and stored at −80 °C. Sema4D concentrations were assessed using an enzyme-labeled immunosorbent assay (ELISA) kit (Xinle Co., Ltd., Shanghai, China) according to the instructions.

### Statistical analysis

SPSS 19.0 software was used to perform χ2 test for categorical variables and t test for continuous variables. The Mann–Whitney test or Kruskal–Wallis test was used to analyze Sema4D levels in serum of MRONJ patients at different time points. Statistically significant differences were indicated by *p < 0.05, **p < 0.01, ***p < 0.001.

## Results

### Demographic and clinical characteristics of patients

The demographic and clinical characteristics of 97 patients who received dentoalveolar surgery were shown in Table 1. A total of 42 of the 97 patients were diagnosed with MRONJ, and the other 55 served as non-MRONJ controls. There were no significant differences in their basic characteristics, including gender, age, duration of BPs use, types of BPs used, and types of surgery. The two groups of patients had osteoporosis and bone metastasis before the use of BPs, and there was no significant difference in the number of patients. In patients in the MRONJ group, the sites of osteonecrosis included the mandible (66.7%), the maxilla (26.2%), and the mandible and maxilla (7.1%). MRONJ grades include stage I (35.7%), stage II (45.2%) and stage III (19.1%).

**Table 1:** Demographic and clinical characteristics of patients received dentoalveolar surgery (n = 97)

Characteristics	Study group		p
	Non-MRONJ (n = 55)	MRONJ (n = 42)	
Gender			
Male	21 (38.2%)	14 (33.3%)	0.674
Female	34 (61.8%)	28 (66.7%)	
Age (years)	65.4 ± 9.6	68.7 ± 10.7	0.108
Duration of bisphosphonate exposure (months)	44.2 ± 27.8	51.1 ± 32.5	0.223
Indication for bisphosphonate treatment			
Osteoporosis	36 (65.5%)	30 (71.4%)	0.661
Bone metastasis	19 (34.5%)	12 (28.6%)	
Administration route			
IV	20 (36.4%)	22 (52.4%)	0.149
PO	35 (63.6%)	20 (47.6%)	
Bisphosphonates use			
Alendronate	12 (21.8%)	8 (19%)	0.212
Pamidronate	3 (5.5%)	1 (2.4%)	
Ibandronate	16 (29.1%)	16 (38.1%)	
Zoledronate	22 (40%)	11 (26.2%)	
Risedronate	2 (3.6%)	6 (14.3%)	
Dentoalveolar surgery			
Tooth extraction	36 (65.5%)	25 (59.5%)	0.765
Dental prosthesis	11 (20%)	11 (26.2%)	
Root cannel procedure	8 (14.5%)	6 (14.3%)	
Location			
Mandible	–	28 (66.7%)	–
Maxilla		11 (26.2%)	
Mandible and maxilla		3 (7.1%)	
Stage			
I	–	15 (35.7%)	–
II		19 (45.2%)	
III		8 (19.1%)	

Values were expressed as n (percentage, %) or mean ± SD. p values for each group were derived from Mann–Whitney test. Chi-square test or Fisher’s exact test was used for assessing distribution of observations or phenomena between two groups

### Serum Sema4D among different groups

**Fig. 2:**
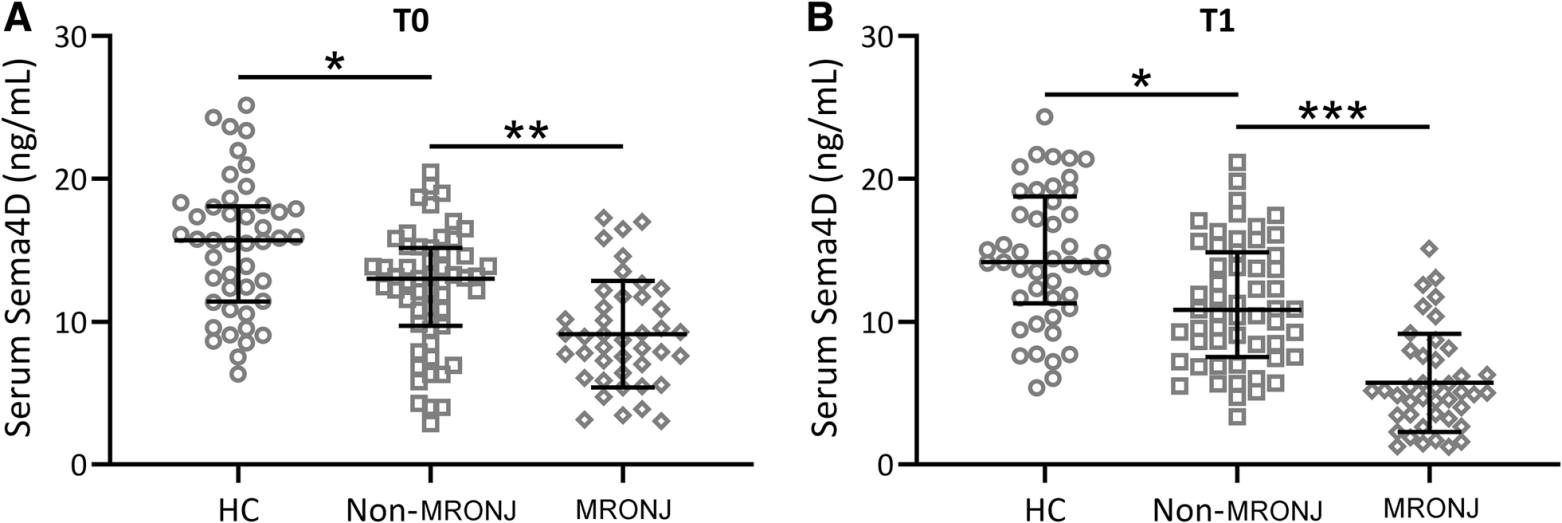
Comparison of serum Sema4D among healthy control, non-MRONJ and MRONJ at enrollment (T0, **A**) and diagnosis (T1, **B**.) Data were presented with median (IQR). *p < 0.05, **p < 0.01, ***p < 0.001. Kruskal–Wallis test followed by Dunn's multiple comparisons test

To further analyze the relationship between the onset of MRONJ and the level of Sema4D in serum, we compared serum Sema4D among healthy control, non-MRONJ and MRONJ at enrollment (T0, Fig. 2A) and diagnosis (T1, Fig. 2B) using Kruskal–Wallis test. As shown in Fig. 2A, the serum Sema4D of patients in the non-MRONJ group was significantly lower than that in the healthy group (p < 0.05). In addition, the Sema4D levels of patients in the MRONJ group were also significantly lower than those in the non-MRONJ group (p < 0.01). As shown in Fig. 2B, the Sema4D level of patients in MRONJ group was significantly lower than that in non-MRONJ group patients after diagnosis in MRONJ group patients, and the difference between the two groups was more significant (p < 0.001).

### ROC analysis of serum Sema4D for the prediction and diagnosis of MRONJ

**Fig. 3:**
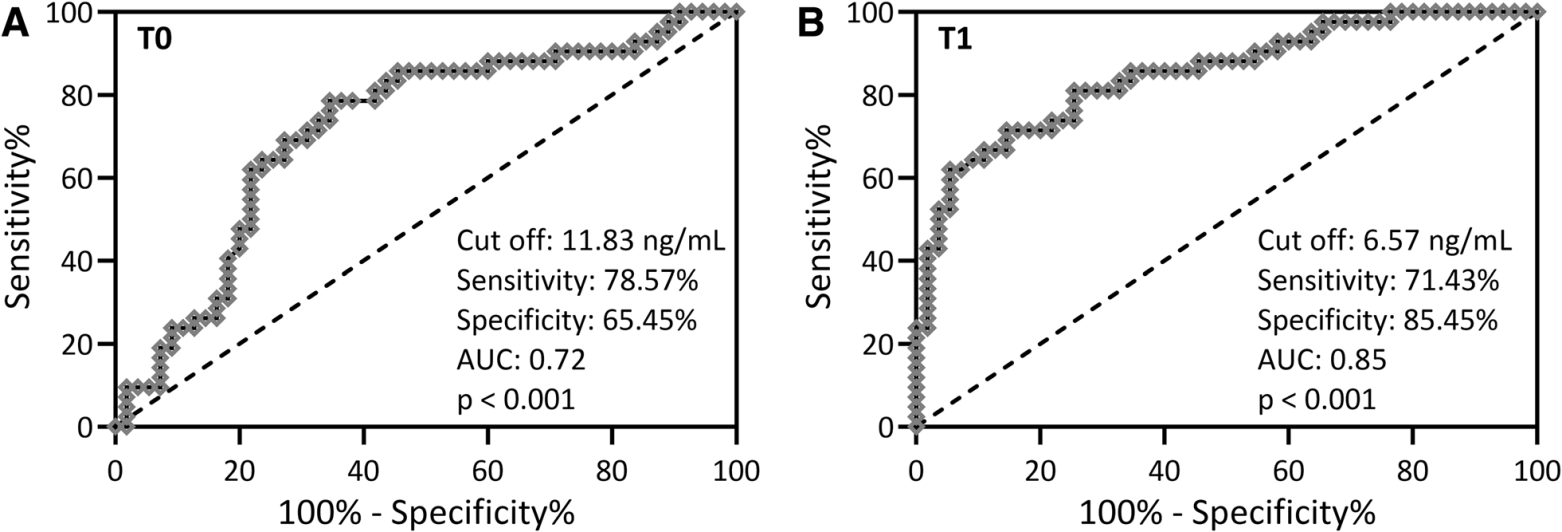
ROC analysis of serum Sema4D for the prediction **A** and diagnosis **B** of MRONJ among patients with the risk of osteonecrosis of the jaw

To further analyze the predictive ability of Sema4D for the onset of MRONJ, we used ROC analysis to analyze serum Sema4D for the prediction (Fig. 3A) and diagnosis (Fig. 3B) of MRONJ among patients with the risk of osteonecrosis of the jaw at T0 and T1. For Sema4D at T0, the cut off value was 11.83 ng/ml, the sensitivity was 78.57%, the specificity was 65.45% and the AUC was 0.72 (p < 0.001, Fig. 3A). For Sema4D at T1, the cut off value was 6.57 ng/ml, the sensitivity was 71.43%, the specificity was 85.45% and the AUC was 0.85 (p < 0.001, Fig. 3B).

### Serum Sema4D in MRONJ patients with different stages and different routes of BPs administrations

**Fig. 4:**
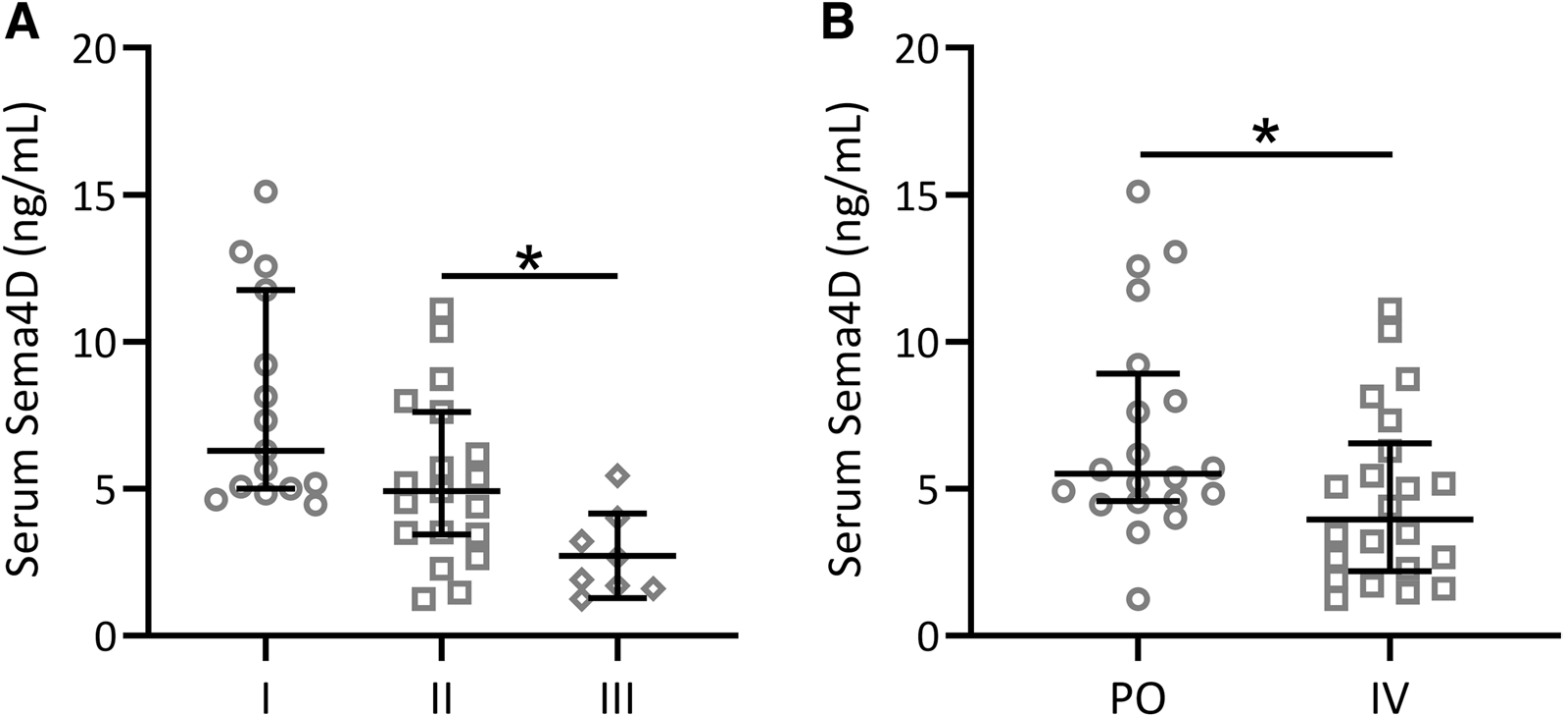
Comparison of serum Sema4D in MRONJ patients with different stage (**A**) and different routes of bisphosphonate administrations (**B**). Data were presented with median (IQR). *p < 0.05. Mann–Whitney test and Kruskal–Wallis test

To further analyze the relationship between Sema4D and MRONJ, we compared serum Sema4D in MRONJ patients with different stages (Fig. 4A) and different routes of bisphosphonate administrations (Fig. 4B). There were significant differences in serum Sema4D levels in patients with grade III MRONJ with increasing disease severity, and there was no significant difference between stage I and II (Fig. 4A). On the other hand, the serum Sema4D level of the intravenous BPs was significantly lower than that of the oral BPs (Fig. 4B).

## Discussion

BPs have been discovered since the 1970s and have been widely used in clinic [18]. BPs were mainly used in clinic to treat osteoporosis and Paget's disease and control the bone metastasis of malignant tumors by inhibiting the function of osteoclasts [19]. BPs mainly affect the formation and activation of osteoclasts by down-regulating the transduction of RhoA signaling channel, and further affecting the cytoskeleton and other structures and the migration of intracellular vesicles [20]. Third-generation BPs, including zoledronic acid and ibandronate, are more resistant to bone resorption [21]. The incidence of adverse reactions of third-generation BPs was significantly lower than that of first- and second-generation BPs [22]. Therefore, now the third generation BPs have been widely used in clinical treatment. Clinical studies have shown that BPs have a very good therapeutic effect on the treatment of bone resorption diseases and the control of bone metastasis [23, 24]. However, it has been found in long-term applications that even the improved third-generation drugs still have a certain degree of adverse reactions and side effects, such as acute phase reactions (gastrointestinal reactions, skin symptoms, etc.) that can appear in a short period of time [22]. More importantly, long-term use of BPs can lead to severe skeletal sequelae, mainly including MRONJ, atypical femoral fractures, and skeletal muscle pain, which seriously affect the long-term quality of life of patients [25].

MRONJ is a rare benign lesion of the jaw in oral and maxillofacial surgery, which is more common in patients with malignant tumors treated with long-term intravenous administration of zoledronic acid [26]. Oral soft tissue in patients with MRONJ is difficult to heal after alveolar surgery. These patients develop osteomyelitis of the jaw several weeks later, with the formation of sequestrum, fistulas, etc. In 2014, the American Association of Oral and Maxillofacial Surgeons (AAOMS) recommended that MRONJ be expanded into medicine-related osteonecrosis of the jaw (MRONJ) [27]. AAOMS believes that in addition to BPs, other antiresorptive drugs such as denosumab, antiangiogenic drugs such as bevacizumab, and sunitinib may also cause osteonecrosis of the jaw [28]. Epidemiological statistics show that the incidence of MRONJ is significantly correlated with the patient's primary disease, the drugs used, the time of administration, the way of administration, and the dosage of medication [29]. For patients with postoperative chemotherapy for malignant tumors, regular intravenous use of zoledronic acid and alveolar surgery, the longer the medication time, the higher the incidence of MRONJ. No effective clinical treatment for MRONJ has yet been found [30]. Therefore, a series of preventive measures such as preventive dental care and timely intervention for early MRONJ symptoms are more important for patients who may develop MRONJ.

In this study, we further verified the clinical value of Sema4D in the early prediction of MRONJ, and provided the basis for the early warning and diagnosis of MRONJ. Since alveolar surgery is a known predisposing factor for MRONJ, we included in this study 97 patients at risk for MRONJ who underwent alveolar surgery at our hospital. After 12 weeks of follow-up, 42 patients were diagnosed with MRONJ, implying that the probability of alveolar surgery leading to the onset of MRONJ with major medication history of BPs is extremely high. We believe that this is because long-term medication of BPs and alveolar surgery are important risk factors for the occurrence of MRONJ, and their combined effects lead to a high incidence of MRONJ in our study population. We measured serum Sema4D levels in all participants at two time points before surgery (T0) and after the end of follow-up (T1). We found that the level of Sema4D in serum of patients with confirmed MRONJ was significantly lower than that of non-MRONJ and healthy controls at two different time points T0 and T1, suggesting that Sema4D may have an inhibitory effect on the occurrence and development of MRONJ. We further performed ROC analysis on Sema4D in both non-MRONJ and MRONJ groups at T0 and T1 time points. We further performed ROC analysis on the predictive effect of participants' serum Sema4D levels on MRONJ diagnosis. Our results show that Sema4D has a significant predictive value for the occurrence of MRONJ.

As a newly discovered family of proteins with the same domain, Semaphorins have bidirectional regulatory effects on osteoclasts and osteoblasts [31]. Factors in the Sema family that have been identified to play a role in bone homeostasis include Sema3A, Sema3E, Sema4D and so on [32]. Sema4D is clearly known to be expressed in osteoclasts. It can act on osteoblasts and can also counteract on osteoclasts [33]. Previous studies have shown that BPs and Sema4D act in opposite ways in many ways. BPs directly inhibited the activity and function of osteoclasts by inhibiting the RhoA and RANKL pathways, thereby reducing jaw bone resorption and increasing bone mineral density [34]. While Sema4D is expressed in osteoclasts, its receptor plexin B1 is expressed in osteoblasts [35]. Sema4D affects the migration of osteoclasts and the migration and functional activation of osteoblasts by activating the RhoA/ROCK pathway [36]. Therefore, it is possible to increase the concentration of sema4D in a cellular environment inhibited by BPs to increase the inhibitory effect of osteoblasts, thereby reducing bone formation. In this study, we also report the mutual exclusion of MRONJ and Sema4D, which is consistent with other findings. We demonstrate that serum Sema4D levels were significantly lower in patients with confirmed MRONJ compared to both non-MRONJ and healthy controls. Furthermore, we found that serum Sema4D levels were significantly lower in patients with grade III MRONJ relative to patients with grade I, II MRONJ. In addition, we reported that MRONJ patients who received intravenous BPs had significantly lower Sema4D levels than those who received oral BPs. This finding is in line with previous studies that patients with intravenous BPs had a higher prevalence of MRONJ than those with oral BPs. Our study to a certain extent indicates that the regulatory effect of Sema4D on bone homeostasis can be affected by BPs, which is related to the occurrence and development of MRONJ. However, the research at this stage is relatively basic, and the clear relationship between Sema4D in the occurrence and development of MRONJ has not been proved. We will continue to focus on this issue in future research.

## Conclusion

In conclusion, we mainly investigated the relationship between serum Sema4D levels and the onset of MRONJ within 12 weeks after dentoalveolar surgery in patients with a history of BPs use in this study. We reported that Sema4D levels were significantly reduced in the serum of patients with confirmed MRONJ. Sema4D has a statistically predictive effect on the occurrence and diagnosis of MRONJ. We hope that our study will provide a rationale for early prevention and intervention of MRONJ.

## Data Availability

All data generated or analysed during this study are included in this published article.

## Abbreviations

BPs: Bisphosphonates
MRONJ: Medication-related osteonecrosis of the jaw
Sema4D: Semaphorin 4D
RANKL: Receptor activator of nuclear kappa-B
ELISA: Enzyme-labeled immunosorbent assay
